# Dr. Newton Graves

**Published:** 1915-02

**Authors:** 


**Dr. Newton Graves,** Western Reserve, 1862, who was formerly in practice at Stafford, died at the Masonic Home at Utica, November 23, aged 86.

---

Of 100 deaths of physicians, many unspecified as to cause, taken serially from the A. M. A. record of the last month, 11 impressed us as especially preventable for men with expert medical knowledge; 3 killed by trains, 2 specified as in automobiles; 1 appendicitis; 3 septicaemia, 2 specified as from wounds during operations; 1 poisoning by toad stools; 1 suicide; 2 typhoid.

